# The Scale for AI Literacy in Health Care Workers: Development and Validation

**DOI:** 10.2196/92373

**Published:** 2026-08-04

**Authors:** Chin-Siang Ang, Sakura Ito, Saumya Bajaj, Minyang Chow, Jennifer Cleland, Jonty Heaversedge

**Affiliations:** 1 Lee Kong Chian School of Medicine, Nanyang Technological University, 11 Mandalay Road, Singapore, 308232, Singapore, 65 69047025; 2 Digital Innovation Office, NHG Health, Singapore, Singapore; 3 Group Clinical Education, NHG Health, Singapore, Singapore; 4 NHG Population Health, NHG Health, Singapore, Singapore

**Keywords:** artificial intelligence, health care workers, literacy, scale development, scale evaluation

## Abstract

**Background:**

AI is increasingly embedded in health care systems; yet, validated instruments for assessing AI literacy among health care workers remain limited. Existing measures are often designed for students or general populations and may not adequately reflect competencies required in health care practice.

**Objective:**

This study aimed to develop and validate the Scale for AI Literacy in Health Care Workers (SAIL-HCW), a new instrument designed to assess AI literacy across domains relevant to health care practice.

**Methods:**

A 3-phase instrument development study was conducted. In Phase 1, conceptual domains were identified through a literature review, and an initial item pool was generated. In Phase 2, content validity was assessed by 4 subject-matter experts, and face validity was evaluated with 26 health care workers. Feedback from both groups informed item refinement. In Phase 3, psychometric testing was conducted using survey data from health care workers in a single health care organization. A total of 425 participants completed the survey. The dataset was randomly split into 2 subsamples for exploratory factor analysis (n=212) and confirmatory factor analysis (n=213). Model fit was evaluated using unidimensional, correlated-factor, higher-order, and bifactor models. Reliability was assessed using Cronbach alpha and McDonald omega. Item performance was examined using corrected item-total correlations (CITC), item discrimination analysis, and inter-item correlations. Construct validity was assessed using prior AI training, frequency of AI use, and self-rated AI literacy.

**Results:**

Phase 2 feedback from experts and health care workers supported the proposed domain structure and informed item refinement, including revision of wording and removal of redundant items. The final SAIL-HCW consists of 14 items across 7 domains, including AI concept, data fluency, AI evaluation, AI in practice, ethics and regulation, AI in system, and continuous learning. In Phase 3, the bifactor model showed the best fit compared with alternative models (comparative fit index and Tucker-Lewis index>0.93; root-mean-square error of approximation<0.06; standardized root-mean-square residual<0.05), indicating a general AI literacy factor alongside domain-specific factors. Internal consistency for the total scale was high (Cronbach α=0.937; *ω*=0.938). Domain-level reliability ranged from 0.635 to 0.797. All items significantly discriminated between high- and low-scoring groups (*P*<.001), with CITC values ranging from 0.570 to 0.785. Construct validity was supported, with higher SAIL-HCW scores observed among participants with prior AI training, higher frequency of AI use, and higher self-rated AI literacy (all *P*<.001).

**Conclusions:**

The SAIL-HCW provides initial evidence of validity and reliability for assessing AI literacy among health care workers. Findings suggest that AI literacy may be represented as a general construct with additional domain-level components. The scale may be useful for research and educational evaluations, although further validation in other settings is required.

## Introduction

AI is increasingly embedded within health systems worldwide, reshaping how care is delivered, managed, and organized [1-3]. The World Health Organization (WHO) defines AI as the “ability of algorithms encoded in technology to learn from data so that they can perform automated tasks without every step in the process having to be programmed by a human” [4]. These systems include a broad range of approaches such as machine learning methods and large language models. AI-based applications are now used across clinical and nonclinical settings to support risk prediction, patient triage, workflow management, and population health surveillance [5,6], influencing both direct care and broader health service organization. Global investment in AI for health continues to rise rapidly, projected to reach US $125 billion by 2028 [7], reflecting strong institutional commitment to its integration into health systems. However, the impact of these technologies is not determined by investment or technical capability alone [8,9]. Their adoption and effectiveness in practice depends fundamentally on how they are understood, interpreted, and used by health care workers in real-world clinical and organizational contexts [10-12].

In this context, AI literacy has emerged as a key component of digital competence in health care [13,14]. AI literacy refers to the knowledge, skills, and attitudes required to understand how AI systems function [15,16], critically evaluate their outputs [11], and apply them appropriately within professional practice [15,16]. It also encompasses ethical awareness and the ability to engage with the organizational and sociotechnical contexts in which AI is deployed [11,13]. Rather than being a fixed competency, AI literacy is increasingly understood as an adaptive capacity that must evolve alongside rapidly changing technologies and clinical applications [17].

Despite its growing importance, empirical evidence on AI literacy among practicing health care workers remains limited. Much of the existing literature focuses on conceptual frameworks, position statements, or policy guidance, with limited attention to measurable competencies in practice [4,18,19]. At the organizational level, AI readiness indices primarily assess infrastructure, governance, and institutional capacity, but provide limited insight into individual-level competence [20]. Yet, it is this self-assessed competence that ultimately determines whether AI tools are used safely, effectively, and appropriately in clinical decision-making [21].

At the individual level, several instruments have been developed to assess AI-related competencies; however, these are not fully suitable for health care workers. The Medical Artificial Intelligence Readiness Scale for Medical Students (MAIRS-MS), for example, is designed for student populations and does not fully capture the applied, context-dependent skills required in clinical practice, such as point-of-care critical evaluation of AI outputs, situational adaptation of AI use across clinical and administrative tasks, adherence to organizational AI policies, and collegial support during AI adoption [22]. Similarly, broader digital health literacy instruments such as the Digital Health Literacy Instrument (DHLI) [23] and the eHealth Literacy Scale (eHEALS) [24] assess general digital competencies but do not adequately address AI-specific capabilities such as algorithmic interpretation, data-driven decision-making, or ethical governance of AI systems. Across existing tools, limitations include restricted target populations, incomplete coverage of AI-relevant competencies, and variable psychometric robustness [25,26]. Collectively, these gaps highlight the absence of a validated, health care–specific instrument for measuring AI literacy among practicing health care workers.

To better define the construct for measurement, a focused synthesis of the AI literacy literature was undertaken. Across disciplines, AI literacy is consistently described as multidimensional, encompassing understanding of AI systems, appropriate use of AI tools, critical evaluation of outputs, and ethical awareness [27-29]. More recent work extends this to include data literacy [30], organizational and contextual understanding [31], and the capacity for continuous adaptation in response to evolving technologies [32]. However, these competencies have not yet been integrated into a single framework tailored to health care practices [33,34].

Drawing on our literature work, 7 interrelated domains of AI literacy in health care were identified: (1) AI concept, (2) data fluency, (3) AI evaluation, (4) AI in practice, (5) ethics and regulation, (6) AI in system, and (7) continuous learning. The AI concept reflects a basic understanding of what AI is, how it works at a general level, and what it can and cannot do [16,21,22,28,35-44]. It provides a foundational frame for interpreting AI outputs, particularly in avoiding overestimation of what AI systems are able to deliver. Data fluency reflects awareness that AI systems are fundamentally shaped by the data on which they are trained [10,21,22,28,36,37,39]. Differences in data quality, completeness, and representativeness influence how systems perform [10,16,22,28,35-37,39,42]. This domain highlights the importance of recognizing that AI outputs are contingent on underlying data structures rather than being inherently neutral or universally applicable.

AI evaluation focuses on the ability to critically appraise AI outputs. This includes judging whether outputs are plausible, recognizing uncertainty, and identifying potential errors or inconsistencies [10,16,22,28,35-37,39,43]. It reflects concerns in the literature regarding overreliance on automated recommendations and the need for continued human judgment when engaging with AI-generated information [21,38,40,44]. AI in practice refers to the application of AI tools within real-world tasks and workflows. This involves selecting appropriate tools, using them effectively, and recognizing when AI is suitable or not suitable for a particular task [10,16,21,22,35,38-43]. It emphasizes the situated and context-dependent nature of AI use rather than purely theoretical understanding.

Ethics and regulation reflect understanding of what is appropriate in the use of AI within professional and organizational contexts. This includes ethical considerations and adherence to relevant institutional or regulatory expectations [10,16,21,22,28,36-38,40-44]. It recognizes that AI use is not value-neutral and must be guided by established ethical and governance standards. AI in system captures an understanding of how AI operates within wider organizational and sociotechnical contexts. AI tools are embedded within workflows, roles, and institutional processes and may influence how work is structured and decisions are made [10,16,28,37,39,42,43]. This domain highlights the importance of viewing AI as part of a broader system rather than an isolated tool. Continuous learning reflects the need for ongoing development of AI-related knowledge and skills. Given the rapid evolution of AI technologies, literacy cannot be considered static. Instead, it requires continual updating and adaptation in response to emerging tools, applications, and changing practice environments [10,38,43]. Together, these domains reflect the cognitive, practical, ethical, contextual, and adaptive competencies required for safe and effective engagement with AI in health care [45,46].

Against this background, this study aimed to develop and validate a new instrument, the Scale for AI Literacy in Health Care Workers (SAIL-HCW). The first objective was to develop a theoretically grounded measure of AI literacy that is relevant to health care practice and reflects the multidimensional nature of the construct. The second objective was to evaluate the validity and reliability of the resulting scale in a sample of health care workers. The resulting scale is intended to support workforce development, education, and policy efforts aimed at strengthening health care workforce preparedness for the competent use of AI in practice.

## Methods

### Study Design

The study was developed through a 3-phase process [47]. Phase 1 involved a literature review to identify conceptual domains of AI literacy relevant to health care workers and to derive initial scale items capturing the identified domains. Phase 2 aimed to evaluate the initial scale items through expert review and pilot testing with 4 experts and 26 health care workers, including assessments of content and face validation. Phase 3 evaluated the validity and reliability of the final scale items quantitatively with 425 health care workers. This included cross-sectional survey research and statistical analysis. This study was reported in accordance with the Consensus-Based Standards for the Selection of Health Measurement Instruments (COSMIN) guidelines for studies on measurement properties [48], and the completed COSMIN checklist is provided as a supplementary file (Checklist 1).

### Participants and Inclusion Criteria

In all stages, participants were required to meet the following criteria: (1) be currently working in a health care–related role, including clinical, allied health, administrative, or ancillary positions; (2) be able to complete an English-language questionnaire; and (3) be willing to provide informed consent. Participants were health care workers employed across acute and tertiary hospitals, specialist services, and community care settings within a major health care provider in Singapore (NHG Health). Additionally, participants needed to be capable of completing study procedures, including expert evaluation or survey participation, as required in each stage.

### Recruitment

Participant recruitment was conducted across multiple institutions within NHG Health using a convenience sampling approach. Data collection for the study took place between March and July 2025. Participants were recruited across Phases 2 and 3 through institutional communication platforms and internal mailing channels. On accessing the survey link, participants were provided with an information sheet describing the study aims, procedures, and data protection measures before providing informed consent.

For Phase 2 (face validation), 26 health care workers were recruited from NHG Health to participate in pilot testing of the scale to assess item clarity and interpretability. Participation was voluntary, and no incentives were provided. Additionally, experts were recruited for Phase 2 (content validation), including 4 experts comprising 1 clinician, 1 AI specialist, and 2 health systems professionals with experience in health care delivery and technology implementation. Experts were independent of the study team, provided informed consent prior to participation, and received no incentives.

For Phase 3 (scale evaluation), the target sample size was determined based on recommended respondent-to-item ratios [49]. For the 28-item version 2 of the SAIL-HCW scale, a minimum ratio of 10 participants per item indicated a minimum sample size of 280, while a preferred ratio of 15 participants per item yielded an ideal target of 420.

### Development of the SAIL-HCW

**Figure 1.**
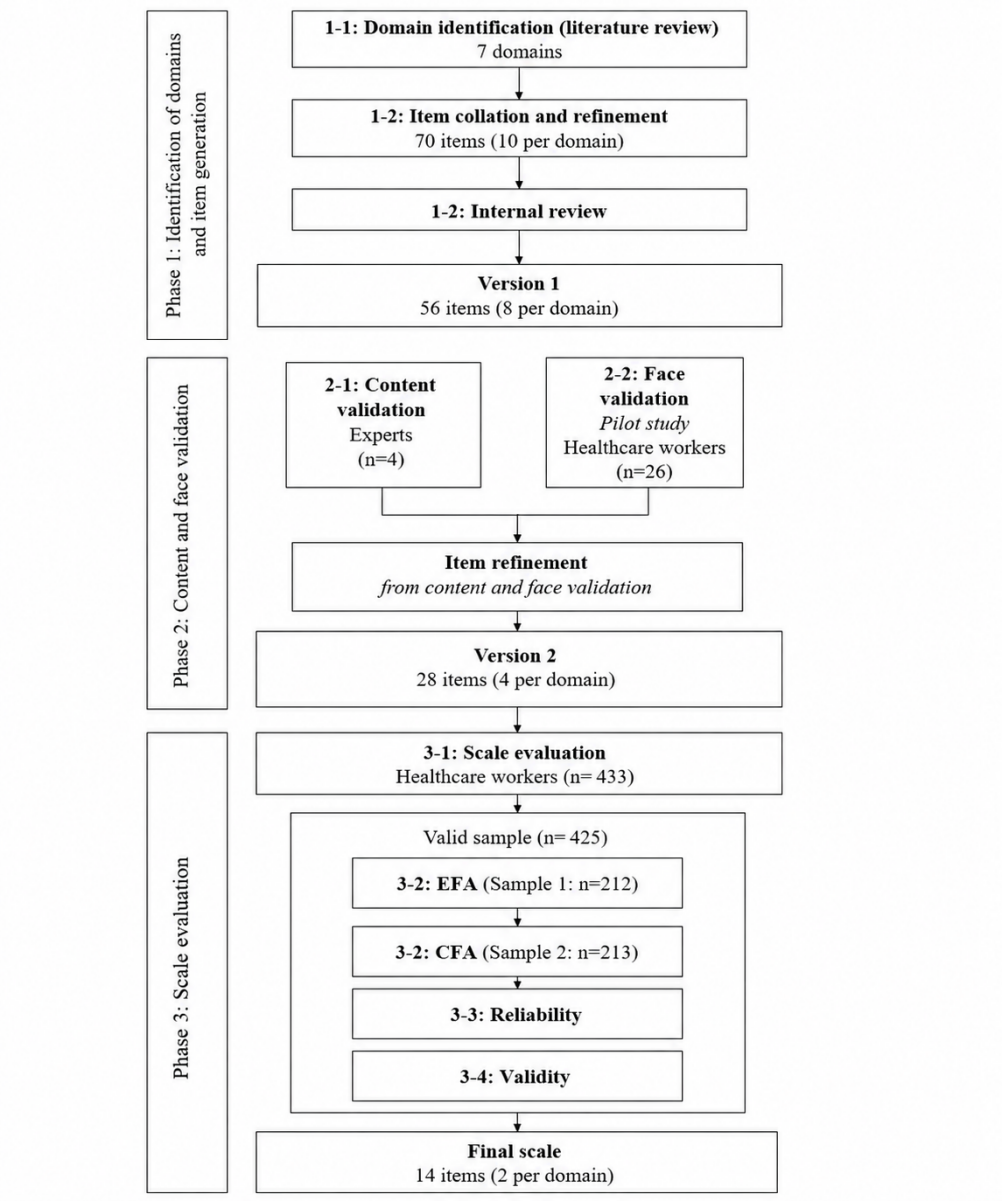
Method phases. CFA: confirmatory factor analysis; EFA: exploratory factor analysis.

The primary objective of this study was to develop and validate a scale to assess AI literacy among health care workers. The instrument was designed to capture key competencies related to understanding, evaluating, and applying AI in health care settings. The overall process of scale development is shown in Figure 1.

### Phase 1: Identification of Domains and Item Generation

#### Phase 1.1: Identification of Domains

A literature review was conducted to identify conceptual domains of AI literacy relevant to health care workers. Searches were performed in PubMed and Google Scholar, focusing on existing studies reporting AI literacy frameworks, scale development, or validation, or descriptions of AI-related knowledge, skills, and competencies. As no health care worker-specific instruments were identified, the search was broadened to include AI literacy literature more generally. The review covered studies published between 2015 and 2024. Study selection and data extraction were conducted independently by 2 authors (CSA and SI), with disagreements resolved through discussion or consultation with a third author (SB).

#### Phase 1.2: Item Generation

Items were generated by the same authors who conducted the literature review, drawing on the domains and subdomains identified in Phase 1‐1 to define the conceptual structure of the scale. Items identified in the literature were mapped to the corresponding domains. During this process, items that were redundant, overly technical, or not applicable to health care settings were removed following assessment of their relevance and appropriateness. An additional internal review was then conducted by 2 authors (MC and JC) who had not been involved in the initial item generation. Each item was independently assessed for conceptual relevance, clarity, and suitability for use with health care workers and categorized as retain, retain with modification, or exclude. Decisions were then discussed and agreed upon. This iterative process resulted in version 1 of the scale.

### Phase 2: Content and Face Validation

#### Phase 2.1: Content Validation

Content validation was conducted with 4 experts to evaluate the relevance and appropriateness of the initial scale items. Data were collected using an online questionnaire administered via Qualtrics (Qualtrics LLC). Experts reviewed the scale items and assessed their alignment with the defined domains using a 4-point Likert scale (1=not relevant to 4=highly relevant). Open-text fields were provided for comments on wording, clarity, and applicability.

#### Phase 2.2: Face Validation

Face validation was conducted with 26 health care workers to assess the clarity and interpretability of the scale items. Participants completed the questionnaire using a 6-point Likert scale (1=strongly disagree to 6=strongly agree). Items were presented in domain-based sections; however, domain names and definitions were not shown, as the aim was to assess interpretability rather than domain recognition. After completing each section, participants were asked to describe, in their own words, what they thought the items were measuring and to identify any items they found unclear, ambiguous, or difficult to interpret. Based on the combined input from content and face validation, the scale was revised to produce version 2 of the SAIL-HCW scale for subsequent psychometric evaluation.

### Phase 3: Scale Evaluation

#### Phase 3.1: Administration of the Scale

An online survey was administered to health care workers using version 2 of the SAIL-HCW scale developed in the previous stage. In total, 433 health care workers were invited to participate, and responses were collected via a secure, government-approved online platform (FormSG; Government Technology Agency). Participants completed the 28-item version 2 of the SAIL-HCW scale, with all items rated on a 6-point Likert scale ranging from 1 (strongly disagree) to 6 (strongly agree). Alongside this, demographic information was collected to characterize the sample, including age, gender, highest educational qualification, primary role, years of work experience, institutional setting, primary department, and patient-facing status. These variables are summarized in Table 1 and were not intended to measure AI-related experience. To examine construct validity [47], 3 AI-related variables were included: prior AI-related training, frequency of AI use at work, and self-rated AI literacy [25,26,50,51]. Training was categorized as none, some, or extensive, while frequency of use was rated from 1 (never) to 5 (every day), and AI literacy from 1 (very low) to 5 (very high). Together, these variables provided indicators of participants’ exposure to and engagement with AI. The survey was kept deliberately concise, with no additional measures included, to maximize feasibility and response rates in busy health care settings [52].

**Table 1.** Descriptive characteristics of participants (N=425).

Characteristic	Participants, n (%)
Age (years), mean (SD; range)	36.17 (9.03; 21-69)
Gender, n (%)	
Men	102 (24.0)
Women	312 (73.4)
Nonbinary	2 (0.5)
Prefer not to say	9 (2.1)
Highest education, n (%)	
Secondary or below	6 (1.4)
Diploma or advanced diploma	45 (10.6)
Professional certificate	12 (2.8)
Bachelor’s degree	254 (59.8)
Postgraduate degree	108 (25.4)
Primary role, n (%)	
Administrative	139 (32.7)
Allied health	86 (20.2)
Ancillary	24 (5.6)
Medical	46 (10.8)
Nursing	94 (22.1)
Pharmacy	36 (8.5)
Years of professional experience, n (%)	
<1	57 (13.4)
1‐5	121 (28.5)
6‐10	81 (19.1)
11‐15	95 (22.4)
16‐20	31 (7.3)
>20	40 (9.4)
Institution, n (%)	
Acute/ Tertiary Hospital	254 (59.8)
Specialty Care Institute	52 (12.2)
Community/ Primary Care Setting	119 (28.0)
Primary department, n (%)	
Direct patient care	126 (29.6)
Clinical support and diagnostic	130 (30.6)
Community, administrative and training	169 (39.8)
Patient-facing, n (%)	
No	173 (40.7)
Yes	252 (59.3)
Formal AI training, n (%)	
None	298 (70.1)
Some (courses/workshops)	117 (27.5)
Extensive (degree/certificate)	10 (2.4)
Frequency of AI use, n (%)	
Never	19 (4.5)
Rarely	106 (24.9)
Monthly	42 (9.9)
Weekly	96 (22.6)
Daily	162 (38.1)
Self-rated AI literacy, n (%)	
Very low	42 (9.9)
Low	188 (44.2)
Moderate	177 (41.6)
High	17 (4)
Very high	1 (0.2)

#### Phase 3.2: Examination of Factor Structure

The factor structure of the scale was examined using exploratory and confirmatory factor analyses to evaluate the underlying dimensional structure of the scale.

#### Phase 3.3: Reliability Analysis and Item Analysis

Reliability was assessed by examining the internal consistency of the scale. Item analysis was conducted to evaluate the performance of individual items.

#### Phase 3.4: Construct Validity

Construct validity was assessed by examining differences in scale scores across groups defined by relevant characteristics, including prior AI training, frequency of AI use, and self-rated AI literacy.

### Data Analysis

Data were analyzed in accordance with the 3 phases of scale development. Each phase involved distinct but complementary analytic approaches aligned with the objectives of item generation, scale refinement, and psychometric evaluation. The analyses for each phase are described below.

In Phase 1, domains and subdomains identified from the literature were analyzed through iterative comparison and grouping of conceptually similar elements. Redundant or overlapping domains were consolidated, and items were mapped to the corresponding domains to ensure conceptual alignment. The process was conducted independently by 2 authors (CSA and SI), with a third author (SB) consulted to resolve discrepancies where necessary. All derived domains and items were then independently reviewed by other team members who had not been involved in the initial scale development process. Each item was assessed for clarity, conceptual relevance, suitability for health care workers, and potential overlap with other items. Feedback from the review was subsequently discussed within the research team before final item refinement.

In Phase 2, content validity and face validity data were analyzed using both quantitative and qualitative approaches. For content validity, item-level content validity indices (I-CVI) were calculated as the proportion of experts rating each item as relevant, and items with I-CVI values below 1.00 were considered for removal [53]. For face validity, item-total correlations (ITC) were calculated to identify items with weak associations with their respective domain scores; items with ITC values below 0.30 were considered for removal [25]. Qualitative analysis for both content and face validity involved reviewing open-ended participant feedback to assess item clarity, interpretability, and relevance, as well as identifying common issues across responses.

Survey data from Phase 3 were analyzed using SPSS (version 26.0; IBM Corp) and AMOS (version 24.0; IBM Corp). The dataset was first reviewed for completeness and response quality [54], and descriptive statistics were used to summarize the sample. Prior to factor analysis, the distributional properties of SAIL-HCW item scores were examined. Univariate outliers were screened using *z* scores, with values outside 3 considered extreme [55]. Skewness and kurtosis were assessed for univariate normality, with values within 2 considered acceptable [55]. Multivariate outliers were assessed using Mahalanobis distance with reference to the chi-square distribution (*P*<.001), and multivariate normality was evaluated using Mardia coefficients for skewness and kurtosis.

Given the logistical challenges associated with conducting research in busy health care settings [52], data were collected at a single time point to minimize respondent burden. A split-sample approach was used to allow independent testing of the factor structure. The dataset was randomly divided into 2 comparable subsamples for separate exploratory and confirmatory factor analyses. Sample 1 was used for exploratory factor analysis (EFA) and Sample 2 for confirmatory factor analysis (CFA). Equivalence between the 2 subsamples was assessed prior to analysis.

EFA was conducted to examine the underlying factor structure and how items clustered without imposing a predefined model, as well as to identify weak or cross-loading items. The suitability of the data for EFA was assessed using the Bartlett test of sphericity and the Kaiser-Meyer-Olkin (KMO) measure. A significant Bartlett test indicates sufficient interitem correlations for factor analysis, while KMO values above 0.70 indicate adequate sampling [56]. EFA was performed using principal axis factoring, with direct oblimin rotation applied to allow for correlations between latent factors. The number of factors to retain was determined using multiple criteria. Eigenvalues greater than 1.0 were used as an initial guide [57], while the scree plot was inspected to identify the point at which the slope of eigenvalues leveled off, indicating diminishing returns from additional factors [58]. Parallel analysis was also used to compare observed eigenvalues with those generated from random data; factors were retained when observed eigenvalues exceeded the corresponding random values [59]. At the item level, criteria for retention included acceptable primary factor loadings and communalities, with items expected to demonstrate loadings on a single factor and communalities of at least 0.40. Secondary loadings ≥0.20 were used as an indicator of potential cross-loading and conceptual overlap across constructs [55].

CFA was subsequently conducted to evaluate the dimensional structure of the SAIL-HCW by comparing empirically and theoretically informed models. Although EFA findings suggested a domain-general factor, the scale had been conceptually developed around 7 theoretically driven domains. To examine these competing representations of AI literacy, 4 alternative measurement models were tested. Model 1 specified a one-factor structure, in which all items loaded onto a single general AI literacy factor. Models 2‐4 reflected the assumption that AI literacy comprises 7 related but distinct domains. Model 2 specified a correlated 7-factor structure, allowing the domains to be associated while remaining conceptually distinct. Model 3 specified a higher-order structure, in which the 7 domains were represented as manifestations of a broader overarching AI literacy construct. Model 4 specified a bifactor structure, in which each item loaded simultaneously onto a general factor, representing AI literacy, and onto its corresponding domain-specific factor, representing more specific competencies. This approach allows evaluation of whether the scale functions as a unidimensional construct, a multidimensional construct, or a combination of both, and provides a more comprehensive assessment of the underlying structure [60]. All models were estimated at the item level without parceling. All analyses used weighted least squares mean and variance adjusted (WLSMV) estimation to account for the ordinal response format [61].

Model fit was assessed using the comparative fit index (CFI) and Tucker-Lewis index (TLI), with values ≥0.90 indicating acceptable fit [62]; the root-mean-square error of approximation (RMSEA) with 90% CI, and the standardized root-mean-square residual (SRMR), with values ≤0.08 indicating acceptable fit [61,63]. Akaike Information Criterion (AIC) values were used to support model comparison, with lower values indicating better relative model fit and greater parsimony [64]. Modification indices were reviewed to identify potential areas of model misfit and to determine whether theoretically justifiable modification could improve model fit. Standardized factor loadings greater than 0.50 were taken as indicative of good item performance [63]. After the factor structure was established, analyses were conducted using the full sample to evaluate item performance, reliability, and validity.

Internal consistency was assessed using Cronbach alpha (α) and McDonald omega (ω) coefficients, which estimate the extent to which items within the scale measure the same underlying construct [65]. Values of ≥0.60 were considered acceptable, and values ≥0.70 were considered good [55]. Item analysis was conducted to evaluate the performance of individual items in terms of their ability to discriminate between respondents and their contribution to the overall scale. Item discrimination was assessed using the 27% upper-lower group method. Total scores were ranked, and respondents in the highest and lowest 27% of the distribution (n=115 in each group) were compared using independent samples *t* tests [47]. Significant differences between groups indicated that items effectively distinguished between higher and lower levels of AI literacy. Corrected item-total correlations (CITCs) were calculated to examine the extent to which each item aligned with the overall scale. Values ≥0.30 were considered acceptable, indicating that items made a meaningful contribution to the construct being measured [55]. Interitem correlations were also examined to assess redundancy and the degree of overlap between items. Values below 0.80 were considered acceptable, indicating adequate discriminant performance between items and no evidence of redundancy [66].

Finally, construct validity was assessed using a known-group approach [47]. SAIL-HCW scores were compared across groups expected to differ in AI literacy based on prior AI-related training, frequency of AI use, and self-rated AI literacy, with higher scores indicating higher levels of AI literacy [25,26,50,51]. For analytic purposes, formal AI-related training was dichotomized into no training versus some or extensive training. Frequency of AI use was grouped as low (never or rarely), moderate (monthly or weekly), and high (daily). Self-rated AI literacy was grouped as low (very low or low), moderate, and high (high or very high). Group differences were examined using independent t-tests or one-way analysis of variance, as appropriate, with statistically significant differences indicating evidence of construct validity.

### Ethical Approval

This study was approved by the Nanyang Technological University Institutional Review Board (IRB-2025‐069). All participants provided informed consent prior to participation. Participation was voluntary, and participants were informed that they could withdraw from the study at any time without penalty by exiting the survey before submission, without providing any explanation. No compensation was provided for participation. To acknowledge participants’ time, respondents were offered the option to enter a prize draw to win one of 20 Singapore $100 vouchers (approximately US $78; SGD $1=US $0.78 as of July 15, 2025). Contact details collected for this purpose were stored separately from survey data and were deleted after completion of the draw. Winners were selected using a random number generator (GraphPad; GraphPad Software LLC), with generated numbers corresponding to participant identifiers. There were no penalties or negative consequences to participants’ employment, benefits, or entitlements if they chose not to participate or withdrew from the study, and any data from participants who withdrew were not processed or analyzed. All data were anonymized and stored securely, with access restricted to authorized members of the research team. Any potentially identifiable information collected during the study was removed after completion of data collection.

## Results

### Phase 1: Identification of Domains and Item Generation

#### Phase 1.1: Identification of Domains

Fifteen studies [10,16,21,22,28,35-44] were identified as key sources for domain development, as they provided explicit descriptions of AI literacy components and sufficient detail to support domain identification. These included framework papers, scoping reviews, qualitative studies, and scale development work. The inclusion of noninstrument studies was considered appropriate given their contribution to describing relevant constructs in the absence of established measurement tools. From the included studies, all content relating to AI literacy domains and subdomains was extracted. In total, 127 domain and subdomain elements were identified. Overlapping or redundant elements were merged through iterative discussion until consensus was reached, resulting in 7 AI literacy domains, including AI concept, data fluency, AI evaluation, AI in practice, ethics and regulation, AI in system, and continuous learning.

#### Phase 1.2: Item Generation

Following domain identification, all extracted descriptors, indicators, and items used in prior studies to represent AI literacy were collated at the item level. This resulted in an initial pool of 317 candidate items. Item refinement was then undertaken to reduce overlap and improve conceptual alignment. Two authors (CSA and SI) independently reviewed the full item pool, identifying redundancy and grouping conceptually similar items. Items that were overly technical or not considered meaningful within health care work contexts were removed. The remaining items were then mapped to the 7 identified domains. Items that could not be clearly aligned with any domain were excluded. This process reduced the pool to 70 items, with 10 items retained per domain to ensure adequate initial content coverage.

Given that most source studies were not developed specifically for health care workers, retained items were further reviewed for contextual relevance. Minor wording adjustments were made where necessary to improve clarity and ensure applicability to health care roles and settings, while preserving the original meaning and domain alignment of each item. All 70 items were then reviewed by the additional internal reviewers who had not been involved in the initial item generation. Based on their feedback, 14 items were removed due to redundancy or insufficient clarity. The remaining items were revised where necessary to improve wording and strengthen domain alignment. This process resulted in version 1 of the 56-item SAIL-HCW scale, comprising 8 items per domain. All items were phrased in English as self-report statements.

Items were formatted using a 6-point Likert scale (1=strongly disagree to 6=strongly agree), selected to maximize response variability and reduce central tendency bias [67,68]. The decision to exclude a neutral midpoint was made to encourage directional responses, as neutrality was not considered a meaningful construct in this context. In addition, previous evidence suggests that even-numbered Likert scales may improve measurement reliability in attitudinal constructs [69].

### Phase 2: Content and Face Validation

Quantitatively, content validation identified 19 items with I-CVI values below the predefined threshold, while face validation identified one item with a low ITC. These items were removed from the scale.

Qualitative feedback from experts was generally positive. Experts supported the conceptual appropriateness and comprehensiveness of the 7-domain structure, with no additional domains proposed. They also highlighted the importance of ensuring balanced item coverage across domains. Similarly, face validation feedback from health care workers indicated that participants’ interpretations were generally consistent with the intended domains, suggesting that the domain structure was intelligible to the target population. Both experts and health care workers also agreed that the 6-point Likert scale was appropriate.

In addition, qualitative feedback identified areas for refinement. Both experts and health care workers noted that 14 items overlapped conceptually with other retained items. These were not retained as separate items; instead, their content was integrated into existing items to reduce redundancy and improve clarity. Health care workers also reported that 4 items containing technical terminology were difficult to interpret. These items were retained, with minor wording revisions made to improve comprehensibility without item exclusion. In response to expert feedback on the need for balanced coverage, 6 new items were added to improve representation across the 7 domains. This process resulted in a version 2 of the SAIL-HCW scale (28-item; see Table 2).

**Table 2.** Definitions of the 7 AI literacy domains and version 2 of the Scale for AI Literacy in Health Care Workers (SAIL-HCW).

Domain	Definition	Code	Item
AI concept	Understanding what AI is, including its basic principles, common applications, and general capabilities and limitations.	C1 C2 C3 C4	“I understand what AI is and can describe it in simple terms.” “I understand how AI technologies, such as machine learning or generative AI, work in a general way.” “I understand the potential benefits and risks of using AI in health care.” I can identify the limitations (eg, bias, black box, and explainability) of AI technologies used in health care.
Data fluency	Understanding how data are prepared and used in AI systems, as well as recognizing factors that may influence data quality or interpretation.	D1 D2 D3 D4	“I understand the structure of data used in AI systems (eg, structured and unstructured data).” “I can identify common quality issues in data used to develop AI models (eg, missing cases, inconsistent formats, and unclear sources).” “I can explain how biased or unrepresentative data may affect AI model performance.” “I can judge whether data are complete and appropriate for use in AI systems.”
AI evaluation	The ability to appraise how well an AI tool performs, including recognizing potential errors, understanding uncertainty in outputs, and judging whether the results are dependable enough for use.	E1 E2 E3 E4	“I can assess whether AI-generated outputs are accurate and clinically appropriate.” “I can judge when AI-generated outputs may require additional verification before being used in care decisions.” “I can identify limitations or biases in the outputs of AI systems (eg, in triage tools and documentation systems).” “I can recognize when AI-generated outputs may be unsuitable for certain clinical decisions or patient contexts.”
AI in practice	Understanding how to use AI tools in daily tasks, including recognizing when AI is appropriate, how to incorporate its recommendations into decisions, and how it fits within practical workflows.	P1 P2 P3 P4	“I can effectively use AI tools relevant to my role.” “I can incorporate AI recommendations appropriately into my work.” “I can choose and use AI tools that are suitable for specific tasks in my work.” “I can adjust how I use AI tools based on the situation or task at hand.”
Ethics and regulation	Awareness of ethical considerations and regulatory requirements associated with using AI in practice.	ER1 ER2 ER3 ER4	“I can use AI in health care in ways that are ethical and responsible.” “I understand health care data privacy and security laws related to AI use.” “I can identify potential ethical risks when using AI in health care tasks.” “I follow institutional policies and ethical guidelines when using AI in my work.”
AI in system	Understanding how AI fits within broader organizational systems, including workflow considerations and implementation factors.	S1 S2 S3 S4	“I understand how AI changes current work processes in my organization.” “I know who to work with when AI tools are introduced in my department or team.” “I can support my team in adjusting to new AI-related changes in our work.” “I am familiar with the basic steps involved in adopting AI tools in our organization.”
Continuous learning	Recognizing the need to remain up to date with developments in AI and to engage in ongoing learning to maintain competence.	CL1 CL2 CL3 CL4	“I regularly reflect on my understanding of AI and identify areas where I need to improve.” “I actively seek opportunities to update my knowledge about AI in health care.” “I adapt how I learn about AI based on new trends and technologies in health care.” “I stay informed about new AI tools and applications that could affect my work in health care.”

### Phase 3: Scale Evaluation

#### Phase 3.1: Administration of the Scale

Of the 433 responses received, 8 were excluded during data cleaning (5 incomplete responses and 3 cases of straightlining [54]). This resulted in a final sample of 425 valid responses included in the analysis. Detailed demographic characteristics are presented in Table 1.

#### Phase 3.2: Examination of Factor Structure

All item-level *z* scores fell within ±3, indicating no extreme univariate outliers. Skewness and kurtosis values were within acceptable limits, suggesting no major violations of univariate normality. No multivariate outliers were identified based on Mahalanobis distance, and Mardia coefficients indicated acceptable multivariate normality. The full dataset was then randomly split into 2 subsamples: Sample 1 (n=212) and Sample 2 (n=213) for EFA and CFA, respectively. Both samples exceeded recommended minimum sizes (N=200) for factor analysis [56], and no significant differences in participants’ characteristics were observed between the 2 samples (see Multimedia Appendix 1), supporting the adequacy of the split-sample approach.

#### EFA

**Figure 2.**
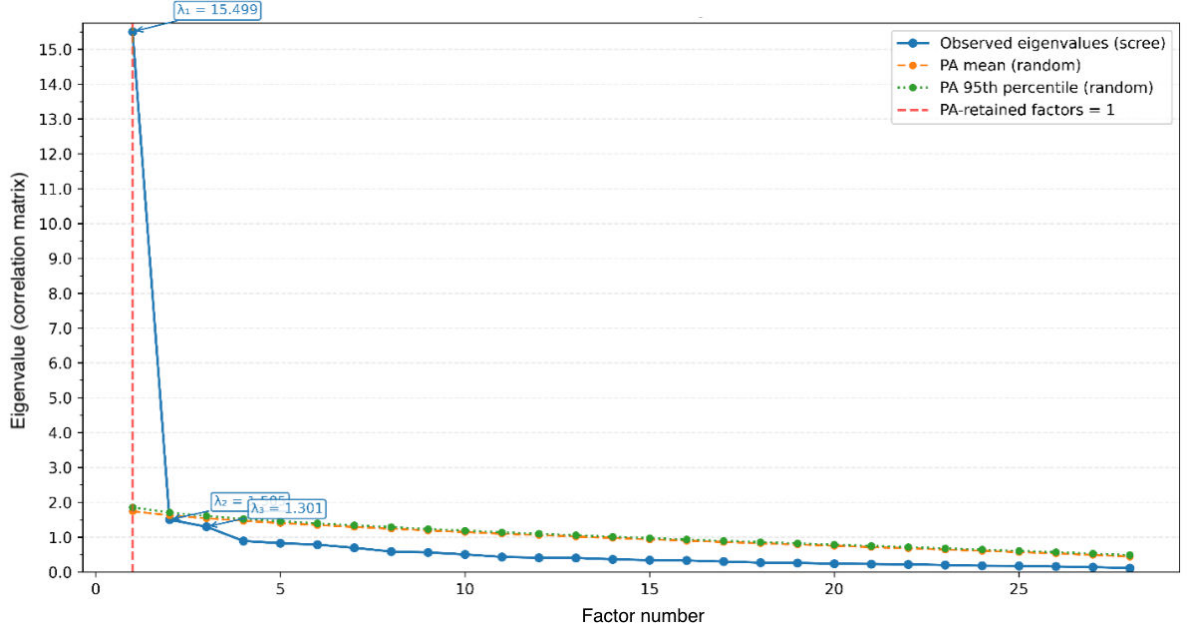
Scree plot of observed eigenvalues overlaid with parallel analysis. PA: parallel analysis.

The dataset demonstrated excellent factorability, with a KMO value of 0.958 and a significant Bartlett test of sphericity (*χ²*_378_=4855.04; *P*<.001). Initial factor extraction identified 3 factors with eigenvalues >1, accounting for 65.38% of the total variance. However, several indicators suggested a predominantly unidimensional structure. The first factor accounted for more than 20% of the total variance [70], and the ratio of the first-to-second eigenvalue was 10.26, exceeding the recommended threshold of 4 [71]. In addition, the scree plot showed a clear inflection after the first factor, and parallel analysis indicated that only the first factor exceeded the 95th percentile of randomly generated eigenvalues, with all subsequent factors falling below this threshold. Together, these results supported retention of a single dominant factor (Figure 2) [72]. At the item level, all items met the predefined criteria for primary loadings (0.597‐0.840) and communalities (≥0.40), with loadings primarily on the first factor. However, 11 items showed secondary loadings ≥0.20, indicating potential cross-loading across constructs [55]. These items were removed to improve interpretability.

Item retention was then reviewed within each conceptual domain. At this stage, 4 domains contained 2 items each, whereas the remaining 3 domains contained 3 items each. To maximize feasibility for use in busy health care settings [73], a further item reduction was undertaken. In domains with 2 items, both were retained; in domains with 3 items, the 2 items with the strongest primary loadings were selected. This resulted in a final 14-item structure covering all 7 domains, including C2, C3 (AI concept); D2, D3 (data fluency); E2, E3 (AI evaluation); P2, P4 (AI in practice); ER3, ER4 (ethics and regulation); S3, S4 (AI in system); and CL2, CL4 (continuous learning). Although EFA indicated a predominantly unidimensional structure, items were retained across all 7 domains in line with the predefined conceptual framework derived from the literature. Results are summarized in Table 3.

**Table 3.** Summary of exploratory factor analysis (EFA). Items in boldface are included in the final scale.

Item	λ^a^			h^2b^
Factor	1	2	3	
C1^c^	0.639	–0.017	0.427	0.591
C2	0.765	–0.078	–0.017	0.691
C3	0.750	–0.101	–0.054	0.576
C4	0.733	–0.272	–0.151	0.612
D1^c^	0.647	0.004	0.508	0.678
D2	0.739	–0.235	0.125	0.616
D3	0.646	–0.267	0.107	0.500
D4^c^	0.831	–0.184	0.316	0.747
E1^c^	0.697	–0.299	0.244	0.635
E2	0.755	–0.374	–0.039	0.711
E3	0.793	–0.308	–0.006	0.724
E4^c^	0.774	–0.281	0.264	0.747
P1^c^	0.738	0.240	0.318	0.704
P2	0.821	0.030	–0.050	0.678
P3	0.814	0.144	0.027	0.683
P4	0.840	0.002	–0.159	0.730
ER1^c^	0.741	0.232	0.021	0.550
ER2^c^	0.717	–0.028	0.220	0.563
ER3	0.755	–0.287	–0.181	0.686
ER4	0.597	0.043	–0.555	0.667
S1	0.748	0.010	0.029	0.580
S2^c^	0.662	0.381	0.058	0.586
S3	0.756	0.194	–0.081	0.670
S4	0.756	0.142	–0.092	0.633
CL1^c^	0.751	0.251	0.072	0.631
CL2	0.731	0.186	–0.022	0.691
CL3^c^	0.785	0.274	–0.114	0.704
CL4	0.788	0.181	–0.139	0.720
Eigenvalues	15.50	1.51	1.30	—^d^
Variance explained (%)	55.35	5.38	4.65	—

a *λ*: standardized factor loadings.

b h2: communality.

c Items with cross-factor loadings ≥0.20 were excluded.

d Not applicable.

#### Confirmatory Factor Analysis

Four competing models were tested: one-factor, correlated 7-factor, higher-order, and bifactor models. Fit indices for all models are presented in Table 4. The one-factor model (Model 1) demonstrated acceptable overall fit, although TLI fell marginally below recommended thresholds. The correlated 7-factor model (Model 2) showed improved fit, suggesting that the domain structure captured additional variance beyond a single general factor. The higher-order model (Model 3) did not improve model fit relative to the correlated model, providing limited support for a strictly hierarchical structure. The bifactor model (Model 4) demonstrated the best overall fit across all indices, with the highest CFI and TLI values, the lowest RMSEA and SRMR, and the smallest AIC. This pattern suggests that the bifactor structure best accounted for both the strong general factor identified in the EFA and the theoretically derived domain structure. Modification indices were examined, but no model adjustments were made, as these did not meaningfully improve model fit.

**Table 4.** Fit indices for alternative Scale for AI Literacy in Health Care Workers (SAIL-HCW) measurement models.

Models	*χ*^2^ (df)		CFI^a^	TLI^b^	RMSEA^c^ (90% CI)	SRMR^d^	AIC^e^
One-factor	237.132^f^ (77)		0.909	0.897	0.071 (0.057-0.085)	0.061	293.132
Seven-factor correlated	143.694^f^ (56)		0.953	0.914	0.064 (0.047-0.081)	0.051	241.694
Higher-order	216.932^f^ (70)		0.917	0.893	0.072 (0.058-0.087)	0.059	286.932
Bifactor	86.837^f^ (50)		0.980	0.936	0.054 (0.035-0.074)	0.042	208.837

a CFI: comparative fit index.

b TLI: Tucker-Lewis index.

c RMSEA: root-mean-square error of approximation.

d SRMR: standardized root-mean-square residual.

e AIC: Akaike Information Criterion.

f *P<*.001.

**Figure 3.**
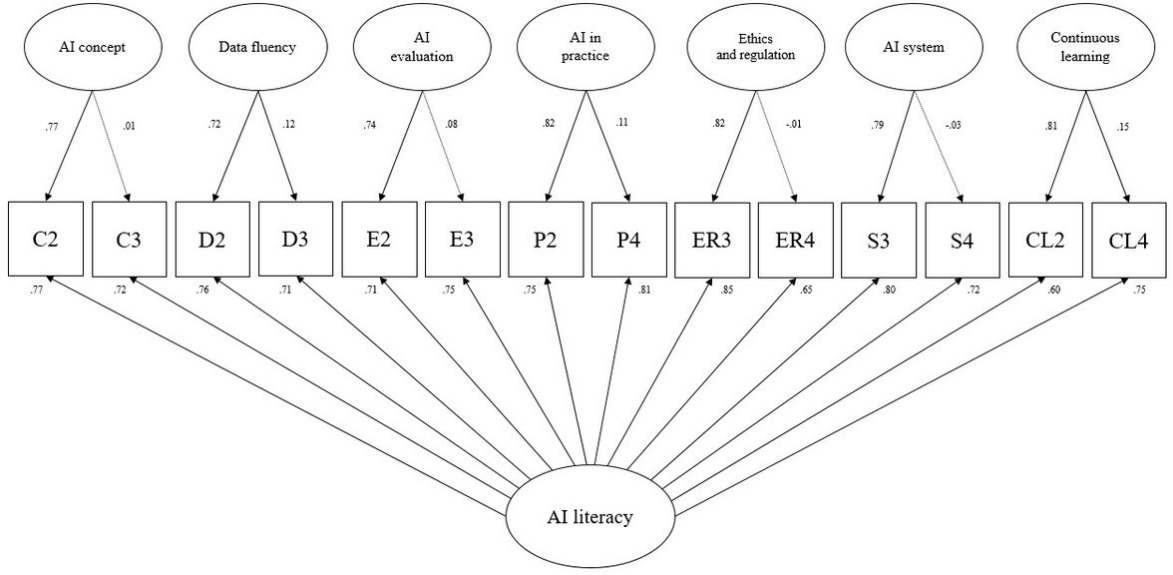
Bifactor model of Scale for AI Literacy in Health Care Workers (SAIL-HCW). Latent variables are represented by ellipses; items are represented by rectangles. Error of measurement is not shown but was specified for each variable in the model. Solid black arrows denote significant pathways, *P*<.05; dashed arrows denote pathways that were not statistically significant.

Examination of factor loadings further clarified the bifactor structure (see Figure 3). For simplicity, factor loadings for the first 3 models are presented in Multimedia Appendix 2, while only the factor loadings for Model 4 are reported in the Results section. All items loaded significantly and positively onto the general AI literacy factor, with loadings exceeding 0.50, indicating substantial shared variance across items. In contrast, loadings on the domain-specific factors were smaller and more variable. For most items (except E2, P2, and CL2), loadings on the general factor exceeded those on the corresponding domain factor, suggesting that the general factor accounted for most of the explained variance. Two items (ER4 and S4) showed negative loadings on their specific factors, likely reflecting crossover suppression effects commonly observed in bifactor models [74].

Overall, these results suggest that although AI literacy was conceptualized as a multidimensional construct, responses to the SAIL-HCW appeared to be driven by a single strong general AI literacy factor. The bifactor model, therefore, provided the most appropriate representation of the data, supporting the use of a total scale score as the primary indicator, while retaining the 7 domains as theoretically meaningful subcomponents rather than independent subscales [60].

### Phase 3.3: Reliability Analysis and Item Analysis

#### Reliability Analysis

Internal consistency was high for the total scale (α=0.937; *ω*=0.938). At the domain level, α and ω values ranged from 0.635 to 0.797, indicating acceptable to good internal consistency across all 7 domains (Table 5).

**Table 5.** Reliability analysis.

Reliability	Total scale	AI concept	Data fluency	AI evaluation	AI in practice	Ethics and regulation	AI in system	Continuous learning
Cronbach α	0.937	0.683	0.704	0.766	0.797	0.635	0.708	0.773
ω	0.938	0.686	0.704	0.767	0.797	0.635	0.708	0.774

#### Item Analysis

Item analysis results are presented in Table 6. All items showed statistically significant differences between the high- and low-scoring groups (*P*<.001), indicating that each item effectively discriminated between respondents with differing levels of AI literacy. CITCs ranged from 0.570 to 0.785, indicating that each item contributed meaningfully to the overall scale. Interitem correlations ranged from 0.287 to 0.662, indicating that items were related but not redundant (see Multimedia Appendix 3). Overall, these findings support the discriminative capacity of individual items and the discriminant validity of the scale.

**Table 6.** Item analysis.

Item	CITC^a^	Total sample, mean (SD)	LG^b^, mean (SD)	UG^c^, mean (SD)	*t* test (df)
C2	0.681	4.04 (1.13)	3.03 (1.07)	5.00 (0.70)	–16.53^d^ (228)
C3	0.681	4.56 (1.00)	3.67 (1.09)	5.30 (0.69)	–13.53^d^ (228)
D2	0.718	3.68 (1.26)	2.49 (1.08)	4.77 (0.86)	–17.69^d^ (228)
D3	0.627	3.92 (1.29)	2.78 (1.21)	4.90 (0.90)	–15.14^d^ (228)
E2	0.703	4.03 (1.26)	2.77 (1.22)	5.00 (0.86)	–16.04^d^ (228)
E3	0.729	3.67 (1.21)	2.46 (0.96)	4.72 (0.84)	–18.99^d^ (228)
P2	0.753	4.09 (1.11)	3.01 (1.06)	5.07 (0.73)	–17.10^d^ (228)
P4	0.785	4.05 (1.14)	2.86 (1.12)	5.07 (0.66)	–18.29^d^ (228)
ER3	0.737	3.98 (1.16)	2.81 (1.05)	4.97 (0.79)	–17.68^d^ (228)
ER4	0.570	4.58 (1.12)	3.74 (1.33)	5.29 (0.72)	–10.96^d^ (228)
S3	0.720	3.87 (1.21)	2.68 (1.04)	4.92 (0.77)	–18.57^d^ (228)
S4	0.682	3.74 (1.23)	2.58 (0.95)	4.82 (0.80)	–19.54^d^ (228)
CL2	0.620	4.04 (1.25)	2.86 (1.09)	4.92 (0.87)	–15.84^d^ (228)
CL4	0.722	3.94 (1.16)	2.82 (1.03)	4.93 (0.85)	–16.91^d^ (228)

a CITC: corrected item-total correlation.

b LG: lower group.

c UG: upper group.

d *P<*.001.

### Construct Validity

Participants with prior AI-related training scored higher on the SAIL-HCW than those without training. Scores also increased with frequency of AI use, with higher scores observed among participants reporting more frequent use. A similar gradient was observed for self-rated AI literacy, with progressively higher SAIL-HCW scores across increasing self-assessed literacy levels. All group differences were statistically significant (Table 7). These findings were consistent with theoretical expectations and support the construct validity of the SAIL-HCW.

**Table 7.** Construct validity.

Variables	Category	Statistic (*P* value)
AI training, n (%)		*t*_423_=–5.80 (<.001)
No (n=298)	54.01 (12.20)	
Yes (n=127)	61.27 (10.92)	
Frequency of AI use, n (%)		*F*_2, 422_=18.59 (<.001)
Low (n=125)	51.37 (12.12)	
Moderate (n=138)	56.13 (10.15)	
High (n=162)	59.93 (12.80)	
Self-rated AI literacy, n (%)		*F*_2, 422_=56.15 (<.001)
Low (n=230)	51.19 (11.98)	
Moderate (n=177)	61.37 (9.62)	
High (n=18)	68.94 (8.71)	

## Discussion

### Principal Findings

This study developed and evaluated the SAIL-HCW, a brief instrument intended to assess AI literacy among health care professionals (see Multimedia Appendix 4). The findings provide preliminary evidence supporting the scale’s structural validity, internal consistency, item discrimination, and construct validity. A notable finding concerned the underlying structure of AI literacy within the sample. Although AI literacy was conceptualized through 7 theoretically distinct domains, the analyses consistently indicated the presence of a dominant general factor. Among the competing confirmatory models examined, the bifactor model provided the best fit to the data, suggesting that AI literacy among health care workers may be understood as an overarching capability that also includes distinguishable domain-related components.

The conceptualization of AI literacy as a multidimensional construct is consistent with previous literature. Existing frameworks commonly describe AI literacy as extending beyond technical knowledge to include competencies related to understanding data, evaluating AI-generated outputs, ethical awareness, practical application, and adaptation to evolving technologies [10,16,21,22,28,35-44]. Drawing on this literature, this study identified 7 domains considered relevant to health care practice, including conceptual understanding of AI systems, data fluency, evaluation of AI outputs, practical application, ethics and regulation, organizational and sociotechnical awareness, and continuous learning. These domains informed the development of the SAIL-HCW and provided a theoretically grounded framework for operationalizing AI literacy within health care settings.

While the 7-domain framework was conceptually useful, the findings suggest that health care workers may not necessarily experience these competencies as fully distinct in practice. Responses across domains were strongly influenced by a common underlying factor, despite evidence of domain-related variation. This pattern may reflect the nature of health care work, where AI-related tasks often involve multiple competencies operating simultaneously. Interpreting AI outputs, for example, may require conceptual understanding, awareness of data quality, ethical judgment, and practical knowledge of clinical workflows at the same time [15,45]. From this perspective, AI literacy may function less as a set of isolated competencies and more as an integrated professional capability shaped by the realities of clinical and organizational practice.

The bifactor findings help clarify this relationship between multidimensionality and unidimensionality. The bifactor model showed better fit than the one-factor, correlated-factor, and higher-order models, indicating that responses to SAIL-HCW items were influenced both by a general AI literacy factor and by smaller domain-specific factors. Most items loaded more strongly onto the general factor than onto their respective domains, suggesting that respondents tended to approach the items through an overall perception of AI literacy rather than through clearly differentiated competency areas. At the same time, the domain-specific factors retained conceptual relevance, indicating that the 7 domains still captured meaningful aspects of AI literacy beyond the general construct [60].

These findings suggest that AI literacy may be conceptualized as multidimensional while remaining sufficiently unidimensional for practical score interpretation [75]. This distinction may be important for both assessment and educational use. The general factor supports the use of an overall SAIL-HCW score as an indicator of perceived AI literacy, which may be useful in workforce assessment and evaluation contexts. At the same time, the 7 domains may still provide useful information regarding relative areas of strength and limitations. For example, health care workers may report greater confidence in practical AI use while demonstrating less familiarity with ethical, regulatory, or data-related aspects of AI. In this sense, the domains may function more meaningfully as educational and developmental categories than as fully independent psychometric subscales.

The findings also contribute to current discussions regarding the measurement of AI literacy in health care settings. Existing instruments have largely focused on students, trainees, or general populations [37-39,41,43]. Although these measures have contributed to the early development of the field, they may not fully reflect the applied and context-dependent nature of AI use in health care practice. Health care workers are required not only to understand AI systems, but also to evaluate outputs critically, recognize uncertainty and limitations, navigate ethical and organizational considerations, and integrate AI into existing clinical and operational workflows [8-11]. The inclusion of domains such as ethics and regulation, sociotechnical awareness, and continuous learning reflects these broader professional expectations and extends beyond the narrower technical focus commonly reported in earlier instruments.

The continuous learning domain may be particularly relevant within health care contexts. Unlike some forms of professional knowledge that remain relatively stable over time, AI technologies continue to evolve rapidly, often more quickly than formal training programs or institutional guidance [17]. AI literacy may therefore involve not only existing knowledge and skills, but also the capacity to recognize limitations in one’s understanding and adapt to emerging technologies and changing practice environments. The inclusion of continuous learning within the SAIL-HCW reflects this more adaptive view of AI literacy and aligns with wider discussions regarding lifelong learning in digitally enabled health care systems.

The reliability and item-level findings provide additional support for the scale. Internal consistency coefficients for the total scale were high, while domain-level coefficients were acceptable given the brief nature of the subscales [55]. All retained items demonstrated meaningful CITCs and differentiated between respondents with higher and lower levels of AI literacy. Known-group comparisons also showed that SAIL-HCW scores varied according to prior AI-related training, frequency of AI use, and self-rated AI literacy. These patterns were consistent with theoretical expectations and provide additional support for the construct validity of the scale [25,26,50,51].

Overall, the findings suggest that AI literacy among health care workers may be understood as an integrated yet multifaceted capability. Although the 7 domains remain theoretically and practically relevant, responses to the SAIL-HCW were primarily shaped by a dominant general factor. The bifactor structure therefore provides one possible way of understanding AI literacy in health care settings, allowing both overall score interpretation and consideration of domain-related variation.

### Strengths and Limitations

#### Comparison With Existing Measures

This study builds on a growing number of instruments developed to measure AI literacy and related constructs, which vary in theoretical focus, target population, and level of contextual specificity.

The AI Literacy Scale (AILS) conceptualizes AI literacy through 4 dimensions, including awareness, usage, evaluation, and ethics [38]. The scale provides a concise structure supported by confirmatory factor analysis and demonstrates good internal consistency. However, it was developed in a general population sample and focuses on broad AI interaction rather than health care–specific competencies. In contrast, the SAIL-HCW was developed with practicing health care workers and includes domains that reflect clinical and organizational contexts, including AI in systems and data fluency, which are not explicitly represented in AILS.

The Scale for the Assessment of Non-Experts’ AI Literacy (SNAIL) questionnaire adopts a 3-factor structure comprising technical understanding, critical appraisal, and practical application [37]. This model provides a clear distinction between foundational AI knowledge and applied skills. Compared with SNAIL, the SAIL-HCW includes additional domains related to ethics and regulation, continuous learning, and system-level integration. These domains extend measurement beyond individual-level technical and evaluative skills to include organizational and adaptive competencies relevant to health care practice.

The scale developed by Yuan et al [39] introduces a more holistic framework that includes individual, interactive, and sociocultural levels of AI literacy. It incorporates constructs such as algorithm influence, user efficacy, and threat appraisal. While this represents a broader conceptualization of AI literacy, the instrument was validated in a general population sample and is not specific to health care settings. The SAIL-HCW differs by embedding system-level and organizational dimensions within a health care–specific framework, with items reflecting professional roles, clinical workflows, and health care governance structures.

The Meta AI Literacy Scale (MAILS) incorporates psychological and meta-cognitive components, including AI self-efficacy and emotion regulation, alongside knowledge-based competencies [43]. This approach emphasizes self-management and behavioral adaptation in relation to AI use. Compared with MAILS, the SAIL-HCW places greater emphasis on applied professional competencies in health care practice rather than general psychological adaptation to AI. The SAIL-HCW also includes data fluency and AI in systems, which are not central components in MAILS.

The MAIRS-MS is the only instrument among these measures developed specifically for a health care–related population [22]. It assesses cognition, ability, vision, and ethics among medical students and focuses on readiness for future AI integration. While MAIRS-MS provides an important foundation for health care AI measurement, it is designed for students rather than practicing health care workers. The SAIL-HCW differs in target population and in its focus on current professional practice, including the application of AI in clinical and administrative workflows, rather than general readiness or anticipated use.

Across these instruments, common domains include AI knowledge, application, evaluation, and ethics. However, variation exists in the extent to which system-level, organizational, and adaptive competencies are included, as well as in target populations and intended use contexts. The SAIL-HCW addresses these differences by combining individual, practical, ethical, organizational, and continuous learning domains within a single instrument designed specifically for health care workers in practice settings [76].

#### Implications

This study provides a structured instrument for assessing AI literacy among health care workers. The SAIL-HCW offers a multidimensional measure that captures competencies across 7 domains, including AI understanding, data fluency, evaluation, practical application, ethics and regulation, system-level awareness, and continuous learning.

At the individual level, the scale could support identification of variation in AI literacy across health care workers. This includes differences in perceived competence across domains, which could help identify areas where additional training or support is required. The domain structure allows for a more detailed profile of strengths and gaps compared with single-score measures. At the educational level, the SAIL-HCW could be used to inform the design and evaluation of AI-related training programs. Baseline assessment can help identify learning needs prior to training, while follow-up assessment could support evaluation of changes in perceived AI literacy over time. This scale could also support curriculum development by highlighting which competencies are less developed across health care staff groups.

At the organizational level, the SAIL-HCW could assist health care institutions in understanding workforce readiness for AI integration [77]. This includes identifying areas where staff may require additional support to engage with AI-enabled systems in clinical or administrative workflows. The inclusion of system-level and continuous learning domains is particularly relevant in this context, as AI tools are increasingly embedded within organizational processes rather than used as standalone technologies [78]. At a broader policy level, aggregated use of the SAIL-HCW may support workforce monitoring and planning in relation to digital transformation strategies [12]. This could be relevant for health systems seeking to evaluate preparedness for AI adoption across different professional groups and settings. Researchers could also use the SAIL-HCW as a primary outcome variable in intervention studies evaluating the effectiveness of AI-focused training programs or as a predictor variable in studies examining associations between AI literacy and clinically relevant outcomes such as technology acceptance, adoption behavior, or patient safety attitudes.

The scale is designed to be brief and feasible for use in applied health care settings. This increases its potential utility in routine evaluation contexts where time and resource constraints limit the use of longer instruments [52,73]. It should nonetheless be acknowledged that the SAIL-HCW measures self-reported perceptions of AI literacy rather than objectively assessed competence. Its use should therefore be considered alongside other forms of evidence rather than as a standalone indicator of capability. Future work examining the relationship between SAIL-HCW scores and observed behaviors, performance on knowledge-based assessments, or actual engagement with AI tools in practice would help to establish the broader utility of the scale and strengthen the evidence base for its applied use.

#### Limitations

Despite these implications, several limitations should be noted. First, participants were recruited from a single health care organization using a convenience sampling approach. This limits the extent to which findings can be assumed to reflect wider health care populations, particularly those working in different systems or professional contexts. This may also have introduced selection bias, including the possibility that individuals with higher levels of technology or AI-related competence were more likely to participate. In addition, the sample included a proportion of administrative health care staff, reflecting the broader application of AI across both clinical and nonclinical roles within health care settings. Future research would benefit from testing the scale across a broader range of settings and occupational groups, and from further examining potential differences between professional subgroups.

Second, the SAIL-HCW relies on self-reported responses, which reflect perceived rather than directly observed competence. While this is common in literacy and competency research, it may not fully capture actual performance in practice. Future validation work could consider combining self-report measures with knowledge-based tests or task-based assessments to provide a more comprehensive evaluation of AI literacy. Third, each domain is currently represented by a small number of items. While this supports brevity, it may also limit the level of precision available for interpreting domain-specific scores. Further refinement of the item pool may help strengthen subscale measurement in future iterations of the instrument. Finally, this study did not examine aspects such as temporal stability, predictive validity, criterion-related validity, or measurement invariance across groups. These represent important areas for further psychometric evaluations, particularly if the scale is to be used for comparison across professional groups or over time.

### Conclusion

This study reports the development and validation of the SAIL-HCW, an instrument designed to assess AI literacy among health care workers. The scale offers an initial structured measure of a construct that is increasingly relevant as AI becomes more embedded in health care delivery and organization. Findings from the psychometric evaluation suggest that AI literacy in this sample can be represented as a general construct, with 7 underlying domains capturing distinct but related aspects of competence. The scale demonstrated acceptable levels of internal consistency and showed expected patterns of item performance and group differences, supporting its use as a preliminary measure for both research and educational purposes. The SAIL-HCW may be useful for identifying variation in AI literacy within health care workforces and for informing the design of training and educational activities. At the same time, further work is likely needed to examine how the scale performs across different health care settings, professional groups, and levels of exposure to AI technologies. As AI continues to evolve within health care systems, tools that support the assessment of workforce capability may help to clarify current levels of understanding and highlight areas where further development is needed.

## Supplementary material

10.2196/92373 Multimedia Appendix 1 Characteristics of participants in Sample 1 and Sample 2.

10.2196/92373 Multimedia Appendix 2 Interitem correlation matrix for the retained items.

10.2196/92373 Multimedia Appendix 3 Confirmatory factor analysis models with standardized weights for (1) the single factor model, (2) the 7-factor correlated model, and (3) the hierarchical model.

10.2196/92373 Multimedia Appendix 4 Final form of the Scale for AI Literacy in Health Care Workers (SAIL-HCW).

10.2196/92373 Checklist 1 COSMIN reporting checklist.

## Abbreviations

AIC: Akaike Information Criterion
AILS: AI Literacy Scale
CFA: confirmatory factor analysis
CFI: comparative fit index
CITC: corrected item-total correlation
COSMIN: Consensus-Based Standards for the Selection of Health Measurement Instruments
DHLI: Digital Health Literacy Instrument
EFA: exploratory factor analysis
eHEALS: eHealth Literacy Scale
I-CVI: item-level content validity indices
ITC: item-total correlations
KMO: Kaiser-Meyer-Olkin
MAILS: Meta AI Literacy Scale
MAIRS-MS: Medical Artificial Intelligence Readiness Scale for Medical Students
RMSEA: root-mean-square error of approximation
SAIL-HCW: Scale for AI Literacy in Health Care Workers
SNAIL: Scale for the Assessment of Non-Experts’ AI Literacy
SRMR: standardized root-mean-square residual
TLI: Tucker-Lewis index
WHO: World Health Organization
WLSMV: weight least squares mean and variance adjusted
