# Supplementary material for: The Scale for AI Literacy in Health Care Workers: Development and Validation
Source: J Med Internet Res. 2026 Aug 4;28:e92373. doi: 10.2196/92373 (PMC13436800; doi:10.2196/92373)
Supplement: Supplementary file 1 [file jmir-v28-e92373-s001.docx]

Appendix 1. Characteristics of participants in Sample 1 and Sample 2

| Characteristic | Sample 1 | Sample 2 | Statistic *(p-*value)) |
| --- | --- | --- | --- |
| **Age (years), mean ± SD (range)** | 36.66± 9.04 (22-69) | 35.68±9.02 (21-60) | *t =* 1.109 (.268) |
| **Gender, n (%)** |  |  | *χ²* = 1.671 (.643) |
| Male | 54 (25.5) | 48 (22.5) |  |
| Female | 151 (71.2) | 161 (75.6) |  |
| Non-binary | 1 (.5) | 1 (.5) |  |
| Prefer not to say | 6 (2.8) | 3 (1.4) |  |
| **Highest education, n (%)** |  |  | *χ²* = 6.606 (.158) |
| Secondary or below | 3 (1.4) | 3 (1.4) |  |
| Diploma/ Advanced diploma | 28 (13.2) | 17 (8.0) |  |
| Professional certificate | 9 (4.2) | 3 (1.4) |  |
| Bachelor’s degree | 120 (56.6) | 134 (62.9) |  |
| Postgraduate degree | 52 (24.5) | 56 (26.3) |  |
| **Primary role, n (%)** |  |  | *χ²* = 1.763 (.881) |
| Administrative | 66 (31.1) | 73 (34.3) |  |
| Allied health | 46 (21.7) | 40 (18.8) |  |
| Ancillary | 11 (5.2) | 13 (6.1) |  |
| Medical | 23 (10.8) | 23 (10.8) |  |
| Nursing | 50 (23.6) | 44 (20.7) |  |
| Pharmacy | 16 (7.5) | 20 (9.4) |  |
| **Years of professional experience, n (%)** |  |  | *χ²* = 3.642 (.602) |
| <1 year | 24 (11.3) | 33 (15.5) |  |
| 1-5 years | 56 (26.4) | 65 (30.5) |  |
| 6-10 years | 45 (21.2) | 36 (16.9) |  |
| 11-15 years | 50 (23.6) | 45 (21.1) |  |
| 16-20 years | 17 (8.0) | 14 (6.6) |  |
| >20 years | 20 (9.4) | 20 (9.4) |  |
| **Institution, n (%)** |  |  | *χ²* = 2.938 (.230) |
| Acute/ Tertiary Hospital | 127 (59.9) | 127 (59.6) |  |
| Specialty Care Institute | 31 (14.6) | 21 (9.9) |  |
| Community/ Primary Care Setting | 54 (25.5) | 65 (30.5) |  |
| **Primary department, n (%)** |  |  | *χ²* = .727 (.695) |
| Direct patient care | 65 (30.7) | 61 (28.6) |  |
| Clinical support & diagnostic | 67 (31.6) | 63 (29.6) |  |
| Community, administrative & training | 80 (37.7) | 89 (41.8) |  |
| **Patient-facing, n (%)** |  |  | *χ²* = 2.076 (.150) |
| No | 79 (37.3) | 94 (44.1) |  |
| Yes | 133 (62.7) | 119 (55.9) |  |
| **Formal AI training, n (%)** |  |  | *χ²* = .870 (.647) |
| None | 151 (71.2) | 147 (69.0) |  |
| Some (courses/workshops) | 55 (25.9) | 62 (29.1) |  |
| Extensive (degree/certificate) | 6 (2.8) | 4 (1.9) |  |
| **Frequency of AI use, n (%)** |  |  | *χ²* = 1.285 (.864) |
| Never | 8 (3.8) | 11 (5.2) |  |
| Rarely | 52 (24.5) | 54 (25.4) |  |
| Monthly | 19 (9.0) | 23 (10.8) |  |
| Weekly | 48 (22.6) | 48 (22.5) |  |
| Daily | 85 (40.1) | 77 (36.2) |  |
| **Self-rated AI literacy, n (%)** |  |  | *χ²* = 3.211 (.523) |
| Very low | 19 (9.0) | 23 (10.8) |  |
| Low | 96 (45.3) | 92 (43.2) |  |
| Moderate | 85 (40.1) | 92 (43.2) |  |
| High | 11 (5.2) | 6 (2.8) |  |
| Very high | 1 (.5) | 0 (0) |  |
