# Supplementary material for: The Scale for AI Literacy in Health Care Workers: Development and Validation
Source: J Med Internet Res. 2026 Aug 4;28:e92373. doi: 10.2196/92373 (PMC13436800; doi:10.2196/92373)
Supplement: Supplementary file 2 [file jmir-v28-e92373-s002.docx]

Appendix 2: Inter-Item Correlation Matrix for the Retained Items

| Item | C2 | C3 | D2 | D3 | E2 | E3 | P2 | P4 | ER3 | ER4 | S3 | S4 | CL2 | CL4 |
| --- | --- | --- | --- | --- | --- | --- | --- | --- | --- | --- | --- | --- | --- | --- |
| C2 | - |  |  |  |  |  |  |  |  |  |  |  |  |  |
| C3 | .523 | - |  |  |  |  |  |  |  |  |  |  |  |  |
| D2 | .628 | .470 | - |  |  |  |  |  |  |  |  |  |  |  |
| D3 | .469 | .484 | .544 | - |  |  |  |  |  |  |  |  |  |  |
| E2 | .521 | .487 | .628 | .503 | - |  |  |  |  |  |  |  |  |  |
| E3 | .533 | .502 | .634 | .651 | .622 | - |  |  |  |  |  |  |  |  |
| P2 | .576 | .534 | .573 | .434 | .624 | .501 | - |  |  |  |  |  |  |  |
| P4 | .545 | .539 | .585 | .520 | .585 | .610 | .662 | - |  |  |  |  |  |  |
| ER3 | .475 | .592 | .550 | .604 | .593 | .641 | .576 | .612 | - |  |  |  |  |  |
| ER4 | .335 | .521 | .366 | .287 | .396 | .380 | .495 | .545 | .466 | - |  |  |  |  |
| S3 | .520 | .493 | .505 | .462 | .462 | .545 | .599 | .650 | .565 | .463 | - |  |  |  |
| S4 | .469 | .471 | .517 | .400 | .458 | .484 | .581 | .610 | .488 | .463 | .548 | - |  |  |
| CL2 | .488 | .496 | .443 | .344 | .415 | .389 | .517 | .489 | .411 | .416 | .553 | .480 | - |  |
| CL4 | .516 | .497 | .478 | .403 | .498 | .512 | .584 | .583 | .530 | .487 | .597 | .656 | .632 | - |
