# Supplementary material for: The Scale for AI Literacy in Health Care Workers: Development and Validation
Source: J Med Internet Res. 2026 Aug 4;28:e92373. doi: 10.2196/92373 (PMC13436800; doi:10.2196/92373)
Supplement: Supplementary file 3 [file jmir-v28-e92373-s003.docx]

Appendix 3: Confirmatory factor analysis models with standardised weights for (a) the single factor model, (b) seven-factor correlated model, and (c) hierarchical model.
