# Supplementary material for: The Scale for AI Literacy in Health Care Workers: Development and Validation
Source: J Med Internet Res. 2026 Aug 4;28:e92373. doi: 10.2196/92373 (PMC13436800; doi:10.2196/92373)
Supplement: Supplementary file 4 [file jmir-v28-e92373-s004.docx]

Appendix 4: Final form of the SAIL-HCW

Please rate the extent to which you agree or disagree with the following statements:

| Code | Item | Strongly disagree | Disagree | Slightly disagree | Slightly agree | Agree | Strongly agree |
| --- | --- | --- | --- | --- | --- | --- | --- |
| C2 | I understand how AI technologies, such as machine learning or generative AI, work in a general way. | 1 | 2 | 3 | 4 | 5 | 6 |
| C3 | I understand the potential benefits and risks of using AI in healthcare. | 1 | 2 | 3 | 4 | 5 | 6 |
| D2 | I can identify common quality issues in data used to develop AI models (e.g., missing cases, inconsistent formats, unclear sources). | 1 | 2 | 3 | 4 | 5 | 6 |
| D3 | I can explain how biased or unrepresentative data may affect AI model performance. | 1 | 2 | 3 | 4 | 5 | 6 |
| E2 | I can judge when AI-generated outputs may require additional verification before being used in care decisions. | 1 | 2 | 3 | 4 | 5 | 6 |
| E3 | I can identify limitations or biases in the outputs of AI systems (e.g., in triage tools, documentation systems). | 1 | 2 | 3 | 4 | 5 | 6 |
| P2 | I can incorporate AI recommendations appropriately into my work. | 1 | 2 | 3 | 4 | 5 | 6 |
| P4 | I can adjust how I use AI tools based on the situations or task at hand. | 1 | 2 | 3 | 4 | 5 | 6 |
| ER3 | I can identify potential ethical risks when using AI in healthcare tasks. | 1 | 2 | 3 | 4 | 5 | 6 |
| ER4 | I follow institutional policies and ethical guidelines when using AI in my work. | 1 | 2 | 3 | 4 | 5 | 6 |
| S3 | I can support my team in adjusting to new AI-related changes in our work. | 1 | 2 | 3 | 4 | 5 | 6 |
| S4 | I am familiar with the basic steps involved in adopting AI tools in our organization. | 1 | 2 | 3 | 4 | 5 | 6 |
| CL2 | I actively seek opportunities to update my knowledge about AI in healthcare. | 1 | 2 | 3 | 4 | 5 | 6 |
| CL4 | I stay informed about new AI tools and applications that could affect my work in healthcare. | 1 | 2 | 3 | 4 | 5 | 6 |

Note. C = AI Concept
